# Modeling the spatial distribution of Chagas disease vectors using environmental variables and people´s knowledge

**DOI:** 10.1186/1476-072X-12-29

**Published:** 2013-05-31

**Authors:** Jaime Hernández, Ignacia Núñez, Antonella Bacigalupo, Pedro E Cattan

**Affiliations:** 1 Laboratorio de Geomática y Ecología del Paisaje, Facultad de Ciencias Forestales y de la Conservación de la Naturaleza, Universidad de Chile, Santa Rosa 11.315, La Pintana, Santiago, Chile; 2 Laboratorio de Ecología, Departamento de Ciencias Biológicas Animales, Facultad de Ciencias Veterinarias y Pecuarias, Universidad de Chile, Santa Rosa 11.735, La Pintana, Santiago, Chile

**Keywords:** Triatominae, Random forests, Remote sensing, *Triatoma infestans*, *Mepraia spinolai*

## Abstract

**Background:**

Chagas disease is caused by the protozoan *Trypanosoma* cruzi, which is transmitted to mammal hosts by triatomine insect vectors. The goal of this study was to model the spatial distribution of triatomine species in an endemic area.

**Methods:**

Vector’s locations were obtained with a rural householders’ survey. This information was combined with environmental data obtained from remote sensors, land use maps and topographic SRTM data, using the machine learning algorithm Random Forests to model species distribution. We analysed the combination of variables on three scales: 10 km, 5 km and 2.5 km cell size grids.

**Results:**

The best estimation, explaining 46.2% of the triatomines spatial distribution, was obtained for 5 km of spatial resolution. Presence probability distribution increases from central Chile towards the north, tending to cover the central-coastal region and avoiding areas of the Andes range.

**Conclusions:**

The methodology presented here was useful to model the distribution of triatomines in an endemic area; it is best explained using 5 km of spatial resolution, and their presence increases in the northern part of the study area. This study’s methodology can be replicated in other countries with Chagas disease or other vectorial transmitted diseases, and be used to locate high risk areas and to optimize resource allocation, for prevention and control of vectorial diseases.

## Background

American trypanosomiasis, Chagas disease, is caused by the hemoflagellate *Trypanosoma cruzi*, and is primarily transmitted to mammal vertebrate hosts through the faeces of hematophagous insects of the subfamily Triatominae [1]. The geographic distribution of triatomine species extends mainly through the Neotropical and Neoartic regions [2]. Triatomine bug species such as *Microtriatoma trinidadensis*, *Eratyrus mucronatus*, *Belminus herreri*, *Panstrongylus lignarius*, and *Triatoma tibiamaculata* are excellently adapted to specialist niches [3]. However, intrusion of human dwellings into sylvan areas has allowed some triatomine species to adapt to the domestic habitat, putting people at greater risk of contracting the disease. This suggests a long evolutionary history, as well as the recent dramatic spread of a few eclectic, domiciled triatomine species. In fact, in Southern Cone countries, the main human Chagas vector, *Triatoma infestans*, is almost exclusively domestic, meaning all stages of life are completed in close proximity to humans in rural or peri-urban environments, increasing the likelihood of human-vector interaction. Peri-domestic populations (those living in surrounding outbuildings, including animal pens and fences) provide a ready re-infesting population following pesticide treatment of houses. Some sylvan populations are interbreeding with domestic populations [4], whereas others seasonally move between houses and the extra-domicile [5], and still others remain in the wild [6]. It is clear that as the development of human settlements continues into previously uninhabited areas, the risk of human infection increases [7]. In addition, migration of people from highly endemic rural areas has brought the disease and vectors to cities [8].

There are four triatomine species in Chile: *Triatoma infestans*, *Mepraia spinolai*, *Mepraia parapatrica* and *Mepraia gajardoi*[9]. Our study zone has only reported the first two species. *Mepraia spinolai* has been described as a mainly sylvatic species, but there are reports of individuals entering houses [10]. Its described habitat is mainly rocky places and rock piles, but has also been found in terrestrial bromeliads [6]. The prevalence of *M. spinolai*, detected by molecular techniques, varies between 42.7% to 76.1% [6,11,12] in sylvatic foci; the few individuals of this species that were captured in dwellings and sent to the National Health Service presented 22.8% of infection in the study area [13]. Human blood has been detected as part of the blood meal of *M. spinolai*[14]. *Mepraia spinolai* is the main vector of *T. cruzi* in the sylvatic cycle of transmission in its area of distribution. However, *Triatoma infestans* is a more efficient vector, compared to *M. spinolai*[15]. The main differences between both species were in their alimentary profile and in their behaviour. They are particularly evident in the activity rhythm - *M. spinolai* is diurnal -, the time that its bite takes- shorter in *M. spinolai* -, and the delay in the defecation - longer in the latter species. These facts would explain its low epidemiological impact regarding human vectorial transmission [16]. *Triatoma infestans* was found recently in sylvatic environments, associated to endemic terrestrial bromeliads in the Metropolitana Region, with a prevalence of 40.9% [6], and rock piles in the Valparaíso Region, with 36.5% of infection [17], along with periodic findings inside human dwellings, which mainly correspond to winged adults; the infection in the study area was reported to be 48.4% [13]. Almost all domestic colonies have been eliminated by the Chagas disease vector control program; in fact, in 1999 vectorial *T. cruzi* transmission to human population was declared interrupted in Chile [18]. Recent reports on human seroprevalence of *T. cruzi* infection in a national health survey indicated 1.8%, 0.9% and 0.7% in Coquimbo Region, Valparaíso Region and Metropolitana Region, respectively [19].

The vectorial disease transmission depends on ecological and environmental parameters of the ecological niche, which defines the ecologic space within which a species can maintain populations without immigration [20]. The understanding of this complex environmental dependency, by means of ecological niche modeling, would help in answering the spatial and temporal issues relevant to domestic transmission control. Given the lack of local data in most of the study zone, there is a need to find predictive models that will allow extrapolation of the actual data of ecological niche to areas that have similar characteristics. Current statistical models can be grouped into two domains: data modeling and algorithm approaches [21]. Data modeling approach starts with assuming a stochastic data model, such as linear or logistic regression which will be fitted and used to both predict what responses are going to be to future input variables and to extract information about how nature is associating the responses with input variables. Algorithm modeling approach focuses on prediction and uses predictive accuracy to validate the models. In general, the latter approach involves a machine-learning algorithm, decision trees or neural networks, to discover associations between point-occurrence data and sets of electronic maps summarizing environmental/ecologic dimensions that may or may not be important in limiting species’ geographic distributions. This methodology will provide distribution predictive models to be subsequently validated with new or independent data [22].

*Triatoma infestans* is very closely associated to domestic and peridomestic structures. The success of this species in particular is related to the ability to efficiently use the available resources in human environments. However, environmental variables do describe their geographical distribution on a regional scale. This indicates that there is an adequate environmental profile to permit the existence of this vector that is not necessarily associated with the availability of human dwellings [23]. Analyzing the relation between the temperature and the population’s intrinsic rate of natural growth (r) of *T. infestans*, even though temperature was not the only climatic variable that limited the growth capacity of this species’ populations, and hence, its geographical distribution, the prediction of the regression model closely matched the known distribution of this species [24]. The relation between geographical distribution and temperature, humidity, precipitation and altitude was shown for some species of Triatominae [25,26].

Usually there is an indirect association between hematophagous insects and vegetation because the plants shelter warm blooded hosts, which are their feeding sources [23]. Vegetation is a variable that includes temperature effects, precipitation and edaphic properties; because of this, it becomes an indicator related to variables that directly influence the demographic processes (mortality, birth rate, dispersion). What frequently occurs is that animal distribution is not associated to the classic distribution patterns of vegetal communities, but to spatio-temporal variability indicators of photosynthetic active biomass, as NDVI, acquired by remote sensors. So, vegetation can be characterized by its spatio-temporal change patterns [27].

Satellite-based remote sensing offers significant benefits for many applications because it provides historical data for comparison and analysis [28]; as in Medical Entomology, where environmental variables obtained by remote sensors can be used to elaborate the predictive models of the geographic distribution of several disease vectors [29,30]. This tool has been applied to study Chagas disease vectors in a few instances, mainly of different species of *Triatoma*[5,20,22].

Our objective was to determine the distribution pattern of *Triatoma infestans* and *Mepraia spinolai* at a regional level, generating a predictive spatial model of their distribution that incorporates quantifiable macro-environmental variables.

## Results and discussion

### Distribution of positive cases

**Figure 1:**
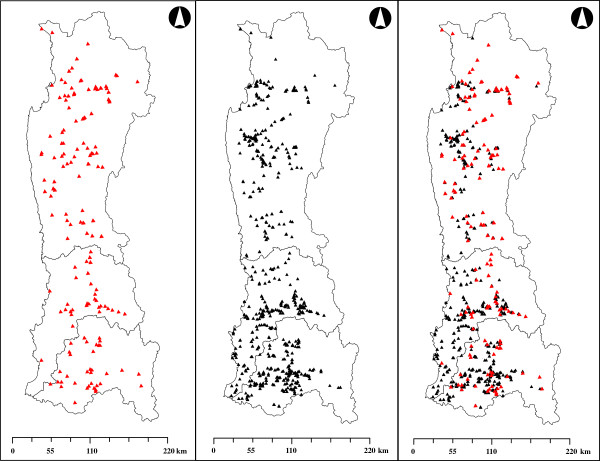
**Spatial distribution of survey results.** Sampling spatial distribution of, from left to right: presences, absences and both.

The results indicated that 14.3% of the rural houses surveyed were positive cases (*i.e.* their inhabitants had seen triatomines). The surveyed houses corresponded to 0.89% of the total rural houses of the study area. Coquimbo Region exhibited the highest value, reaching 24.8% of surveyed dwellings, while Valparaíso and Metropolitana Regions showed lower similar values of 8.8% and 8.5%, respectively (see Table 1). Figure 1 shows the spatial distributions of positive and negative cases. Positive cases increased northwards of the study zone, which supports the previous knowledge that these triatomines prefer habitats with higher temperatures, low rainfall, and xeric vegetation.

**Table 1:** Results of the survey of rural houses regarding triatomines according to people’s knowledge, by region

**Region**	**Rural houses sampling size**	Rural houses positive cases*	**% positive rural houses**
Coquimbo	452	112	24.8
Valparaíso	420	37	8.8
Metropolitana	425	36	8.5
Total	1,297	185	14.3

* The term “positive case” refers to a house in which an inhabitant answered positively regarding having seen triatomines.

In some rural areas we observed individuals of *M. spinolai*, which were found by inhabitants in their homes. This indicates that, despite being a predominantly wild species, it also lives near or within human settlements. Therefore, the habitat of *M. spinolai* and *T. infestans* maybe overlapping more than expected, a situation that reveals the advantage of studying both species together and not separately. This apparently new invasion process of *M. spinolai* to dwellings and peridomicile areas might be related to previous subnotification of its intrusions and therefore lack of scientific reports; however, it is not the first time to be reported [10,13], and it could also be showing the effects of possible reduction of animal preys in their natural environment. In fact, several authors have observed that starved triatomines are more likely to be attracted by live bait [42].

### RF predictions

**Figure 2:**
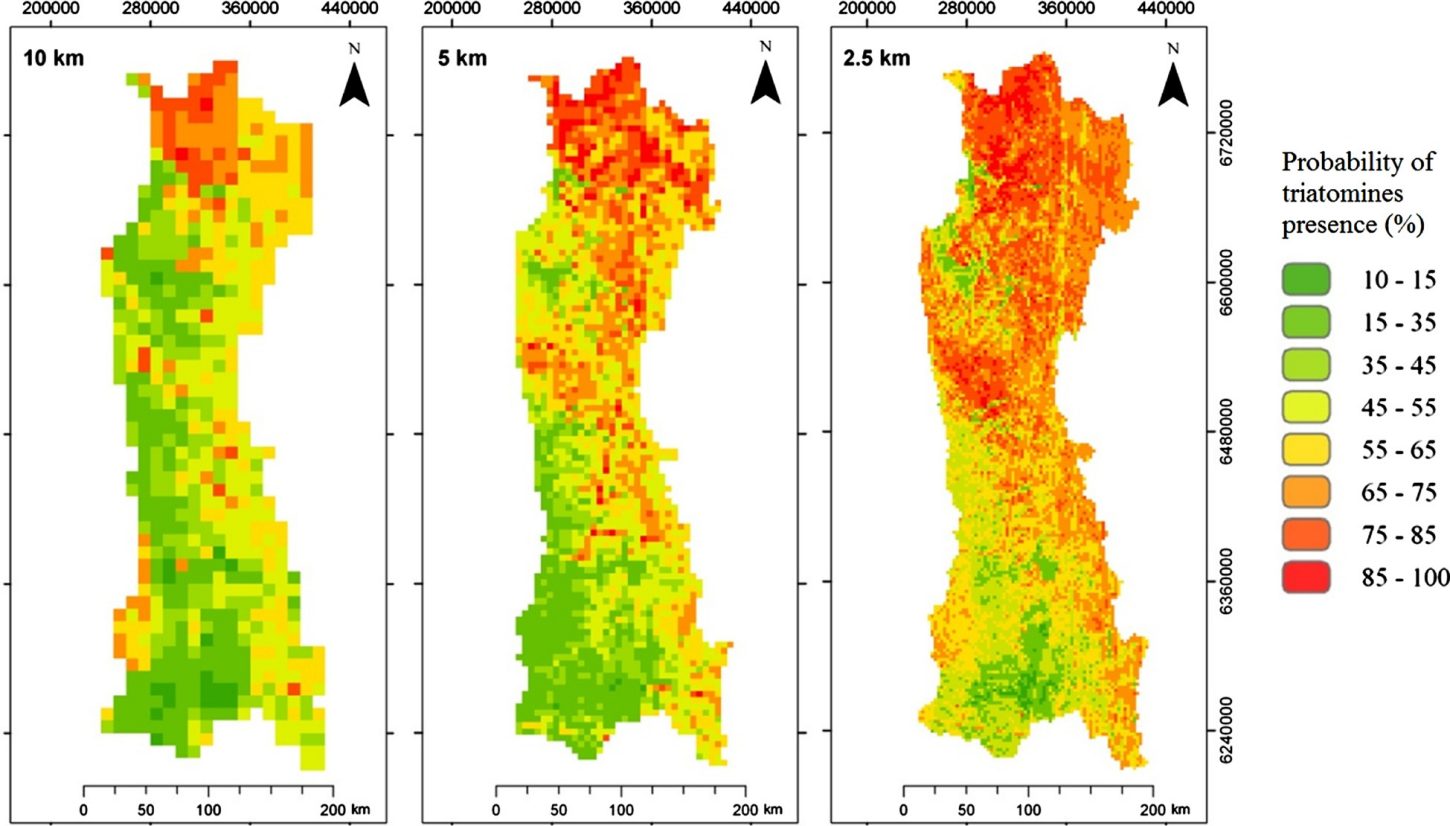
**Predicted triatomines’ presence probability.** Predicted maps of triatomines’ presence probability for 10 km, 5 km and 2.5 km cell size grids, from left to right, respectively.

This is the first study that applies RF to model ecologic niche of vectors of Chagas disease. This approach delivers strong information predictors, and ensures convergence if they are iterative, giving them good predictive accuracy [21]. The five km cell size grid explained more variability than the other tested scales (Table 2). This model exhibited a Pseudo R^2^ of 41.82%, a Mean Square Error (MSE) of 500.47 and a Root Mean Square Error (RMSE) of 22.38. Figure 2 shows the predicted maps of presence probability for each scale.

**Table 2:** Random forests model statistics for each of the cell size grids

**Cell size**	**10 km**	**5 km**	**2.5 km**
**Pseudo R**^**2**^**(%)**	28.79	41.82	14.23
**MSE**	520.25	500.47	749.57
**RMSE**	22.80	22.38	27.37

**Figure 3:**
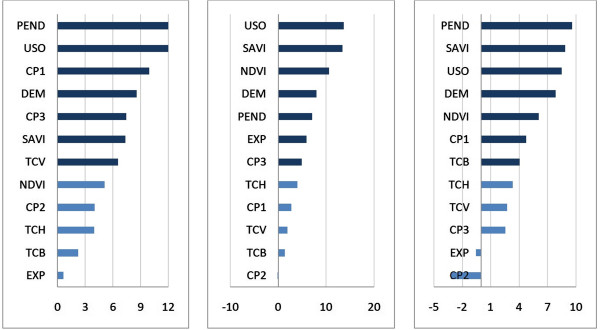
**Predictor variable importance plot.** Variable importance plot generated in randomForest indicating the relative importance of the predictor variable given the increase in the mean square error (MSE) when extracting a variable percentage. The most important variables are shown in dark blue, from left to right, for: 10 km, 5 km and 2.5 km cell size grids, respectively.

The most relevant set of variables (Figure 3) for the 5 km cell size grid model (best) ordered from most to least important are: land use (USO), vegetations indexes (SAVI and NDVI), topography descriptors (DEM, PEND and EXP) and the third principal component (CP3). It is noteworthy that the three most important variables are related to vegetation, confirming its suitability to predict the spatial distribution of triatomines in the landscape, same as described by Gorla for *T. infestans* in Central and South America [23].

Regarding the principal components of the climate layers, for the models 10 and 2.5 km cell size, CP1 showed the greatest importance (Figure 3), which can be explained taking into account that the seasonal temperature is one of the main factors that define the geographical habitat of triatomines [23]. However, for the 5 km cell size grid model, CP3 was more important, showing that rainfall patterns are one of the main climatic characteristics that differentiate places with or without triatomines in the study zone. This was reported previously by Gorla *et al.*[24], who stated that precipitation regime is also an important variable to predict the distribution of *T. infestans* on its own but also in combination with type and altitude distribution of the vegetation.

### Improving the model

**Figure 4:**
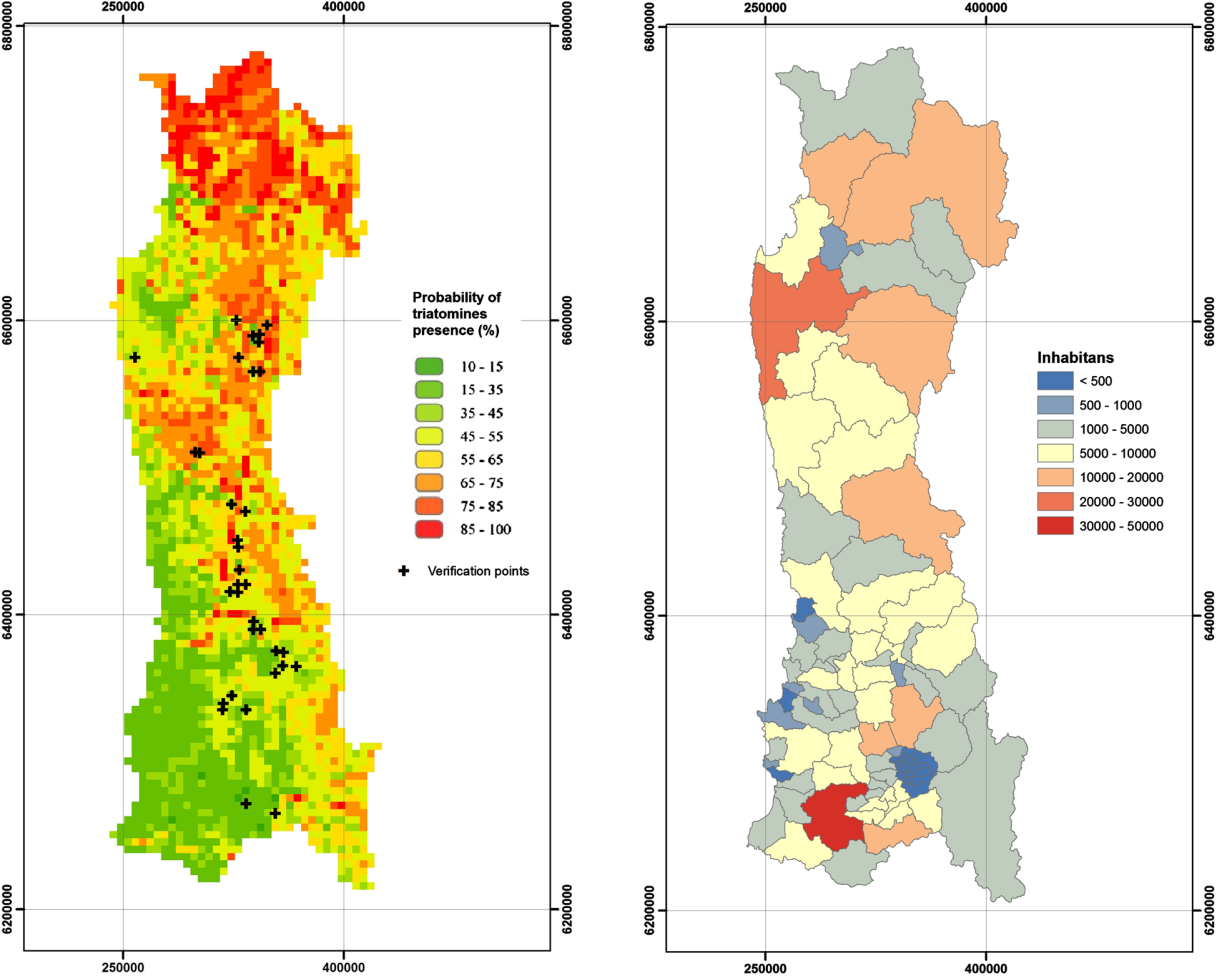
**Prediction map using best predictors, and map of rural inhabitants.** Left: Predicted map of triatomines presence probability for 5 km cell size grid using the most important RF predictors (Pseudo R^2^: 46.2%). Right: Map of number of rural inhabitants in National Survey Districts.

Taking the 5 km cell size grid results as a base, we improved the model by only including the most important variables in a new model of RF algorithm, from most to least important: USO, SAVI, NDVI, DEM, PEND, EXP and CP3. Table 3 and Figure 4 show the results for this new model. They produced a Pseudo R^2^ of 46.2% and a RMSE of 21.5.

**Table 3:** Random forests model statistics for the 5 km cell size grid using the most important variables

**Parameter**	**Value**
Type of random forest	Regression
Number of trees	500
Pseudo R^2^	46.2%
MSE	460.006

According to these results, Coquimbo Region has the highest probability of positive cases for the study zone, with an arithmetic mean of 61.3% of the surface. The Metropolitana Region, on the other hand, has the lowest overall probability with an average of 36.9% of the surface. Table 4 resumes values for all three regions.

**Table 4:** Average probability of triatomines presence (PbbP) according to the best model for each region of the study zone

**Region**	**PbbP**	**Assessed area (km**^**2**^**)**	**Actual area (km**^**2**^**)**
Coquimbo	61.3	31,627	40,580
Valparaíso	39.0	13,797	16,396
Metropolitana	36.0	11,814	15,403

The best estimation, that explains 46.2% of the spatial distribution of triatomines, coincides with the distribution reported for *T. infestans* in Latin America, on a continental scale [23,43-45]. They established that the spatial distribution of *T. infestans* in Chile increases from the center (Metropolitana Region) towards the north. Similarly, in our model, presence probability distribution increases towards the north (Coquimbo Region), tending to cover the central-coastal region and avoiding areas of the Andes range.

On the other hand, there is a tendency towards the south to have lower probabilities of presence by the coast, near the Pacific Ocean. Regarding the tendency of the genus *Mepraia* being located away from the coast in its southern distribution, our results seem to be in agreement with those of Frías [9]. For *M. spinolai*, there were no other previous studies on ecologic niche modeling. Therefore, this prediction is the first approach to establish its spatial distribution.

Knowing the probable location of these species will allow the sanitary authorities to be able to optimize the resource distribution, allocate more in areas of greater risk, and less in those with minimum probability. It will also allow them to include areas that at present are not incorporated in the Chagas disease prevention and control programme, but are suspected to have triatomines according to this prediction, aiming to keep the country free of the vector-borne Chagas disease transmission.

The same approach applied to gather the data for this study - surveying rural population, educating them regarding Chagas disease and its vectors, and obtaining information about triatomines presence or absence - could be applied in other countries where Chagas disease or other vectorial illnesses are present, combined with ecologic niche modeling, to provide a base map to be used for prevention and control of these diseases, for resource optimization; and it can also be used as base for other studies, to predict locations of vectors on a smaller scale: a local model, to more accurately predict where triatomine foci are [46].

## Conclusions

This investigation is the first approach to model the spatial distribution of vectors of Chagas disease in an endemic area using the methods described. The methodology proposed, which included a survey, environmental variables and ecological niche modeling, was successfully used for this end. Triatomines had higher probability of presence in the northern part of the study zone. However, the amount of population at risk in the southern area makes it equally important, even with lower probabilities of triatomines. The best results were found using 5 km cell size grids, and smaller resolutions could not improve the results. The relationship between organisms and their environment are one of the most important causes of spatial distribution patterns of species, allowing their description to be reasonably well explained by climatic factors on broader spatial scales. However, at smaller scales it is likely that the species distribution responds to factors such as resource allocation and micro-environmental variations. This can be explained by assuming the existence of a different set of variables which express its influence on more detailed scales. We believe that a better model should incorporate both regional and local predictors in order to fully understand triatomines’ spatial distributions. We recommend developing similar studies on higher scales, focusing the analysis on the local level, allowing a more accurate definition of the interactions between these species and environmental variables. This study will be a tool to optimize resource allocation, for those working in prevention and control of Chagas disease, and to keep the country free of the vector-borne Chagas disease transmission.

This study could also be used as an example of the use of people’s knowledge regarding a particular vectorial disease to be modelled, combined with environmental variables using the tools provided. For example, by using the free software environment R-project to obtain a prediction for that disease in this or other countries.

## Methods

### Study design and sample

**Figure 5:**
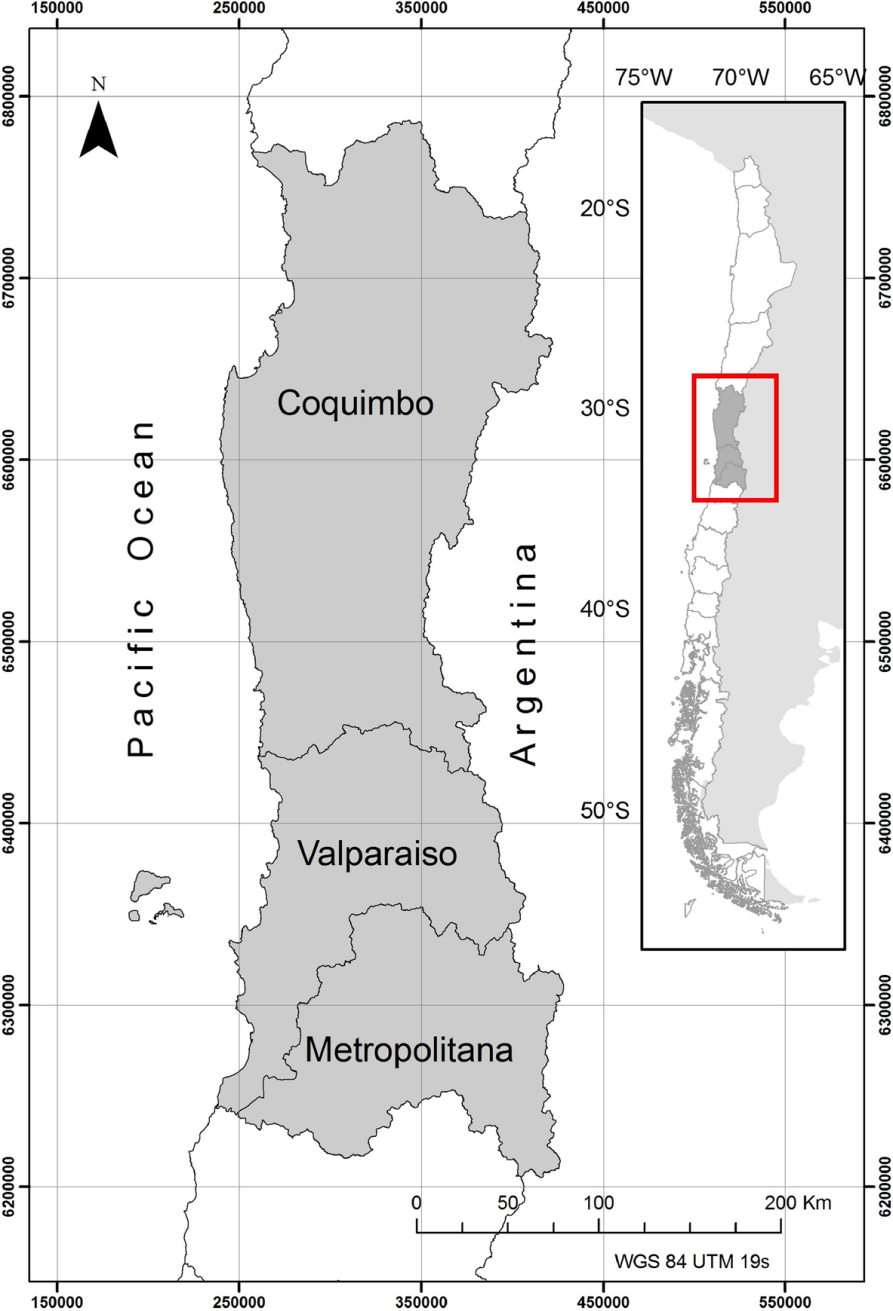
**Map of the study zone in Chile.** Map of the three regions of the study zone: Coquimbo, Valparaíso and Metropolitana (central Chile).

The study zone corresponds to north-central Chile: Coquimbo Region, Valparaíso Region and Metropolitana Region, comprising a total area of approximately 72,300 km^2^ (Figure 5). In order to obtain indirect field data for presence/absence of triatomines - *Mepraia spinolai* and *Triatoma infestans* - we surveyed rural areas belonging to the study zone. To validate the information reported by the householders, firstly we used two sets of pictures with unlabelled images of the target species and other insects, to check if they were able to identify triatomines among the other insects. Afterwards, we used laboratory samples of *T. infestans* and *M. spinolai* in different developmental stages (eggs, nymphs, adults) to ensure a correct identification and to gather further data about whether they had seen these insects, inside or outside their dwellings. The participants’ answers were registered to the survey of that dwelling.

The statistical design of the surveys was a stratified random sampling using clusters of three rural houses as a sampling unit. We divided each of the three regions into their communes, and then into National Survey Districts (NSD). Using data from the Chilean National Statistics Institute a sampling size of about 1% of the total number of rural houses was assigned to each commune. Then, within each commune, the sampling units were allocated to NSD, in proportion to their sizes in terms of their total rural houses. This way, a few NSD from each commune were selected for sampling. Table 5 shows the sampling size and proportion in each region.

**Table 5:** Sampled number of rural houses and total rural houses in each region

**Region**	**Samples**	**Total**	**%**
Coquimbo	452	48,702	0.93
Valparaíso	420	43,521	0.97
Metropolitana	425	54,256	0.78
Total	1,297	14,6479	0.89

### Environmental variables

As input to model the probability of presence of triatomines 12 environmental variables, grouped in four sets, were used.

Group 1 Climate layers from the WorldClim database (http://www.worldclim.org) gridded to 1 km^2^ resolution. We performed a Principal Component Analysis PCA [31] to the 19 available variables to summarize the information into a smaller number of components and we used only the first three components (CP1, CP2 and CP3). From the principal components analysis of these variables, the first component (CP1) was found highly related to seasonal mean temperature, while the second (CP2) and third (CP3) components were mainly related to the thermal variation and rainfall patterns, respectively.

Group 2 LANDSAT TM data images, from 2009 and 2010, were obtained from the Earth Explorer Web site of the United States Geological Survey (USGS). Subsequently, a geometric correction was performed using polynomial rectification based on Chilean regular cartography 1:50000 with 30 control points per image, obtaining a Root Mean Square Error (RMSE) less than 30 m. Also, standard radiometric corrections were applied on all images to reduce the atmospheric effect following the method proposed by Chavez [32], and for the topographic correction the one proposed by Riaño *et al.*[33]. Once the images were corrected, a set of vegetation indexes were calculated: Normalized Difference Vegetation Index NDVI [34], and the Soil Adjusted Vegetation Index SAVI [35,36]. Finally, tasseled cap components [37] Brightness (TCB), Greenness (TCV) and Wetness (TCH), were obtained.

Group 3 The NASA/NGA Shuttle Radar Topography Mission SRTM digital elevation model (90 m) was freely downloaded from the site Earth Explorer. Slope (PEND), aspect (EXP) and altitude (DEM) were directly calculated from SRTM data. Only areas under 3,500 m above sea level were included.

Group 4 Land cover map, scale 1:50000, obtained from CONAF-CONAMA-BIRF National datasets [38]. We used these layers to classify every land cover type into habitat suitability scores (USO) for triatomines using assuming no habitat as 0% and perfect habitat as 100% (Table 6). This determination was made by national experts on triatomines’ biology, using scientific literature review as a complement.

**Table 6:** Classification of land cover types (USO) into habitat suitability scores for triatomines

**Land cover**	**Habitat suitability score (%)**
Open shrubs and succulent plants	90-100
Dense shrubs and succulents	80-90
Open shrubs	70-80
Dense shrubs with/without trees	60-70
Rock formations	50-60
Open land and urbanizations	40-50
Steppes and second growth forests	30-40
Prairies and forests	20-30
Forests and dense plantations	10-20
Other land covers	< 10

### Data grids construction

In order to obtain appropriate data sets for further analysis regular grids for the whole study zone were built. As it was not known which size was going to best suit, 10, 5 and 2.5 kilometers as alternatives cell sizes (100, 25 and 6.25 km^2^) were used, respectively. Using these grids, the average and standard deviation of the 12 environmental variables for each cell were extracted. To add presence/absence to all cells containing sampling units, the following probability was assigned:

$$P=\frac{\mathrm{Positive}\_\mathrm{cases}}{\mathrm{Positive}\_\mathrm{cases}+\mathrm{Negative}\_\mathrm{cases}}*100$$

Where, *P* is the probability of triatomines presence (PbbP), *Positive_cases* and *Negative_cases* are the number of houses with positive and negative identification of triatomines by the householders, respectively. So in each cell of the three grids the following 12 variables were finally obtained: DEM, PEND, EXP, NDVI, SAVI, TCB, TCV, TCH, CP1, CP2, CP3 and USO.

### Random forests predictor

As prediction was this study’s focus, preference was given to the algorithm modeling approach to model the triatomines spatial distribution. Random Forests algorithm (RF) was used to predict the probability of triatomine presence (PbbP), using the set of 12 environmental variables as input. In the RF [39], implemented in the R-project package *randomForest*, prediction is obtained by amassing regression trees each constructed using a different random sample of the data, and then choosing splits of the trees from subsets of the available predictors, randomly chosen at each node. The RF models in this study were obtained by amassing 500 trees as base classifiers, with 12 variables tried at each split. The main result of this procedure is a presence probability map of triatomines in the study zone.

### Validation

For each run, RF gives the associated Mean Square Error (MSE), thus not requiring cross-validation or some form of independent validation to obtain an estimation of the model error [40]. From this, the RMSE, simple statistic that measures the precision of each RF model was calculated [41].

The results obtained using the best model were compared with an independent dataset consisting of positive houses, which were determined as such by direct collection of triatomines performed by trained personnel of the National Health Service in 34 grid cells of our study area. This data is presented in Figure 4 as verification points (black crosses). According to the model, for the same 34 cells, the average probability of presence is 51.9% with a standard deviation of 16.3%. In other words, the model is able to predict the actual presence of triatomines in the study area.

## Competing interests

The authors declare that they have no competing interests.

## Authors’ contributions

JH made substantial contributions to conception and design, supervising the data processing and statistical analysis; he also wrote this manuscript. IN obtained the survey data, processed spatial data and made statistical analyses, with posterior interpretation of the data. AB contributed to the survey design, interpretation of results, writing and editing of this manuscript, giving final approval of the version to be published. PC made contributions to conception of this study and critical revision of important intellectual content of this manuscript; he has also given final approval of the version to be published. All authors read and approved the final manuscript.
